# Systemic inflammatory ratios and their added value to cytology in cervical cancer detection: a multicenter study

**DOI:** 10.3389/fmed.2026.1802477

**Published:** 2026-05-13

**Authors:** Gabriella Vajda, Lotti Lőczi, Balázs Vida, Petra Merkely, Barbara Sebők, Ferenc Bánhidy, Nándor Ács, Richárd Tóth, Balázs Lintner, Márton Keszthelyi

**Affiliations:** 1 Department of Obstetrics and Gynecology, Semmelweis University, Budapest, Hungary; 2 Maternity Obstetrics and Gynecology Private Clinic, Budapest, Hungary; 3 Workgroup of Research Management, Doctoral School, Semmelweis University, Budapest, Hungary

**Keywords:** biomarkers, cervical cancer screening, conization, cytology–histology discrepancy, lymphocyte-to-monocyte ratio, neutrophil-to-lymphocyte ratio, risk stratification, systemic inflammation

## Abstract

**Background:**

Cytology-based screening is central to cervical cancer prevention but may underestimate invasive disease. Low-cost adjunctive biomarkers could improve triage, particularly in settings with limited access to advanced diagnostics. We evaluated cytology–histology concordance and the utility of systemic inflammatory indices in women undergoing cervical conization.

**Methods:**

This multicenter retrospective study analyzed 1.142 women who underwent conization between 2021 and 2024 across three gynecological departments in Budapest, Hungary. Cytological and histological findings were grouped into four clinically relevant grades. Preoperative neutrophil-to-lymphocyte (NLR), platelet-to-lymphocyte (PLR), and lymphocyte-to-monocyte ratios (LMR) were derived from routine blood counts and assessed using non-parametric tests, receiver operating characteristic (ROC) analysis, logistic regression, and Bayesian updating.

**Results:**

Cytology underestimated histological severity in 13.9% of cases and missed 79.6% of invasive cancers (sensitivity 20.4%). NLR was significantly associated with histological outcomes and invasive cancer (*p* = 0.032), whereas LMR was associated with cytological severity (*p* = 0.0009). Although discriminatory performance was modest (area under curve (AUC) 0.57–0.60), all markers showed high negative predictive values (>96%). Elevated NLR independently predicted invasive cancer (odds ratio (OR) 2.04, *p* = 0.036), with cancer prevalence increasing across NLR risk strata.

**Conclusion:**

Complete blood count (CBC)-derived inflammatory markers, particularly NLR and LMR, may support cervical cancer triage strategies as low-cost adjuncts with rule-out potential; however, their limited discriminatory performance precludes their use as standalone diagnostic tools. They may still contribute to risk-adapted decision-making, especially in resource-limited settings, but require further prospective validation within multimodal strategies.

## 1 Introduction

Cervical cancer ranks as the fourth most common malignancy among women globally, with the highest incidence observed between 30 and 35 years of age (1). In 2022, it accounted for approximately 662.000 new cases and nearly 349.000 deaths worldwide, and both the incidence and mortality rates of cervical cancer show an inverse association with the Human Development Index (HDI) (2). The high incidence and mortality of cervical cancer are particularly alarming given its pathophysiology: persistent infection with high-risk human papillomavirus (HPV) is the primary cause, accounting for over 95% of cases, and the disease progresses through identifiable stages of cervical intraepithelial neoplasia (CIN1–CIN3), which can be effectively treated if detected early (3).

The conventional approach for cervical cancer screening has been cytology, commonly known as the Papanicolaou (Pap) test or smear. When cytology results indicate abnormalities, the diagnosis is confirmed through colposcopy, and appropriate treatment decisions are guided by biopsies of suspicious lesions for histological analysis.

Loop electrosurgical excision procedure (LEEP) conization is a commonly used technique for treating cervical intraepithelial neoplasia (CIN), providing both diagnostic and therapeutic value by removing abnormal tissue. The findings indicate that, in a substantial proportion of cases, LEEP conization reveals lesions of greater severity than those predicted by initial cytological results, thereby highlighting the limitations of conventional cytological screening methods (4, 5).

Despite advances in HPV-based screening and emerging triage technologies, accurate risk stratification remains challenging, and cytology may underestimate clinically significant disease in a subset of patients. In settings where access to advanced triage tools is limited, inexpensive adjunctive markers could help guide management decisions (6). Extensive research has elucidated the role of individual blood cell populations in oncogenesis: neutrophils promote tumor progression through the secretion of pro-tumor cytokines and growth factors, contributing to immunosuppression and metastasis; platelets facilitate tumor cell invasion and angiogenesis; lymphocytes are central to antitumor immune responses, with reduced lymphocyte counts reflecting impaired immune surveillance; and monocytes can differentiate into tumor-associated macrophages (TAMs), which further support tumor growth and immune evasion (7). Systemic inflammatory indices derived from routine complete blood counts, such as the neutrophil-to-lymphocyte ratio (NLR), platelet-to-lymphocyte ratio (PLR), and lymphocyte-to-monocyte ratio (LMR), have shown prognostic relevance across multiple malignancies, including gynecologic cancers (8–10).

However, their potential utility for preoperative triage and risk stratification in women undergoing conization has not been sufficiently characterized in large, multicenter cohorts.

Therefore, the aim of this multicenter retrospective study was to evaluate cytology–histology concordance and investigate associations between NLR, PLR, and LMR with cytological and histological outcomes, including invasive cancer. The objective was to assess their potential prognostic value and applicability to supporting personalized management strategies in cervical cancer care.

## 2 Materials and methods

### 2.1 Patients

This multicenter retrospective observational study evaluated a cohort of 1,142 patients as part of the SCOPE study (Semmelweis University Conization and Inflammation Outcomes with Predictive Evaluation). The investigation involved an extensive review of medical records collected between 2021 and 2024. A detailed dataset was assembled, including sociodemographic characteristics, gynecological history, clinical assessments, and laboratory findings to provide a deeper understanding of patient outcomes after conization.

**Figure 1:**
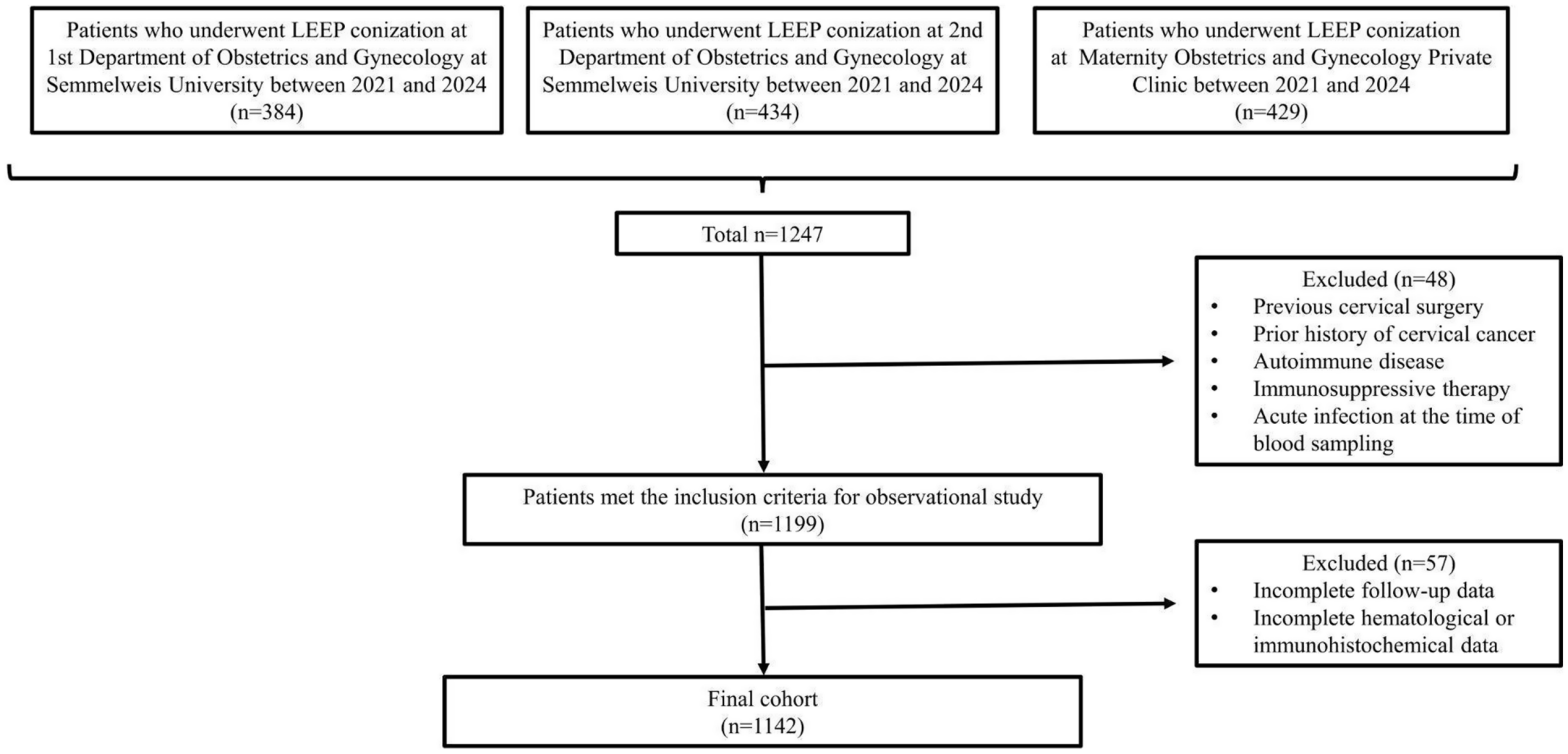
Flowchart of patient selection. The diagram presents the sequential process of including and excluding participants in the study, beginning with the initial screening and progressing to the final eligible cohort, based on clinical, cytological, and histological criteria. Clinically, this illustrates the rigorous selection process ensuring a reliable study population for evaluating cytology, histology, and inflammatory markers.

An initial pool of 1,247 patients who underwent conization in three gynecological centers in Budapest—the 1st and 2nd Departments of Obstetrics and Gynecology of Semmelweis University, and the Maternity Obstetrics and Gynecology Private Clinic—was screened. To be included, patients needed to have undergone conization for cervical dysplasia and possess complete clinical and laboratory data. Exclusion criteria encompassed any previous cervical surgery, a prior diagnosis of cervical cancer, autoimmune disorders, immunosuppressive treatment, acute infections or inflammatory conditions at the time of blood sampling, and incomplete follow-up or hematological information. After applying these criteria, 1,142 patients remained for the final analysis. Figure 1 illustrates the patient selection process.

### 2.2 Characteristics

Sociodemographic variables included patient age, calculated by subtracting the year of birth from the year of surgery. Additional recorded variables comprised body weight, height, body mass index (BMI), and smoking status.

All laboratory measurements were obtained within one month prior to surgery as part of standardized preoperative evaluation, and were performed in facilities accredited by the National Accreditation Authority of Hungary, ensuring compliance with established quality and accreditation standards. No systematic differences in sampling timing were observed between outcome groups. Inflammation-related laboratory parameters, including absolute neutrophil, lymphocyte, monocyte, and platelet counts, were assessed. The neutrophil-to-lymphocyte ratio (NLR) and lymphocyte-to-monocyte ratio (LMR) were calculated from the absolute counts of neutrophils, lymphocytes, and monocytes, whereas the platelet-to-lymphocyte ratio (PLR) was computed as the platelet count divided by the lymphocyte percentage reported in the complete blood count panel.

Cervical dysplasia assessment incorporated cervical cancer screening results and HPV status, with particular emphasis on high-risk HPV types. Based on screening outcomes, patients were assigned to four categories: Grade 1 for negative results, Grade 2 for low-grade squamous intraepithelial lesion (LSIL) and atypical squamous cells of undetermined significance (ASC-US), Grade 3 for high-grade squamous intraepithelial lesion (HSIL), atypical squamous cells, cannot exclude HSIL (ASC-H), atypical glandular cells (AGC), and adenocarcinoma *in situ* (AIS), and Grade 4 for cancer. Surgical data included conization results and definitive histopathological assessments, with excised specimens classified according to the same grading scheme (Grade 1: negative; Grade 2: LSIL and ASC-US; Grade 3: HSIL, ASC-H, and AIS; Grade 4: cancer).

This unified four-grade system was applied to both cytological and histological findings to ensure methodological consistency and comparability across diagnostic modalities. Importantly, the categorization was guided by a risk-based clinical framework rather than purely morphological distinctions. In line with the 2019 risk-based management consensus guidelines of the ASCCP (11), clinical decision-making in cervical screening is primarily driven by estimated CIN3 + risk. Accordingly, low-grade abnormalities such as ASC-US and LSIL, which carry comparable immediate CIN3 + risk and similar management recommendations, were grouped together, whereas high-risk abnormalities associated with substantially elevated CIN3 + risk and the need for prompt intervention were categorized separately. This grading system therefore reflects clinically meaningful risk stratification consistent with contemporary international guidance, reduces diagnostic variability, and prevents statistical underrepresentation of smaller subgroups. Given the limited number of invasive cancer cases (*n* = 53), fully disaggregated modeling of all original cytological and histological subcategories would have resulted in sparse cell counts and unstable parameter estimates; applying the unified four-grade severity system preserved statistical stability in multivariable analyses (12).

This study was ethically approved by the Institutional Review Board of Semmelweis University (SE RKEB: 195/2024).

### 2.3 Data management

For this retrospective study, data were collected and systematically entered into a dedicated database developed for the SCOPE project. This database included comprehensive patient information, such as sociodemographic characteristics, clinical variables, and laboratory results. Prior to analysis, the dataset underwent rigorous quality control, including detailed checks for inconsistencies and outliers, supported by box plot visualizations to identify extreme values that might influence the findings. Any missing information was handled in accordance with predefined procedures to ensure the integrity and reliability of the dataset for statistical analysis. Missing data were handled using a complete-case approach, with analyses restricted to patients who had available data for the variables included in each specific model. No formal imputation methods were applied.

### 2.4 Statistical analysis

All statistical analyses were performed using IBM SPSS Statistics version 25.0 (Released 2017; IBM Corp., Armonk, NY, USA). Continuous variables, including NLR, PLR, and LMR, were nonnormally distributed and are therefore presented as medians with interquartile ranges (IQR). Categorical variables are expressed as frequencies and percentages. Differences in NLR, PLR, and LMR across cytological and histopathological outcome groups (Grades I–IV based on conization results) were assessed using the nonparametric Kruskal–Wallis test. The Mann–Whitney U test was employed to compare laboratory values between groups, particularly to evaluate differences in these markers according to the presence of cancer in histopathological findings.

Receiver operating characteristic (ROC) curve analysis was conducted to evaluate the ability of preoperative NLR, PLR, and LMR to identify invasive cervical cancer. The area under the ROC curve (AUC) was calculated to assess the discriminatory performance of each marker in differentiating patients with invasive carcinoma (histopathological Grade IV) from those with pre-invasive lesions or negative findings. Following established standards, AUC values below 0.60 were considered indicative of poor discrimination, limiting direct clinical applicability despite statistical significance. Optimal cutoff values for NLR, PLR, and LMR were derived from ROC analysis using the Youden index and the cutoff nearest the top-left point of the curve.

For NLR, patients were further stratified into low-, intermediate-, and high-risk groups based on these cutoff values, and the distribution of cancer prevalence across these risk groups was analyzed to assess its clinical relevance. To avoid dependence on arbitrarily defined cutoffs, NLR was also examined by quartiles.

Bayesian probability updating was applied to quantify how NLR modifies the probability of invasive cancer in patients with high-grade cytology. The pre-test probability (prior) was defined as the observed prevalence of invasive cancer within this subgroup (5.15%). Likelihood ratios were derived from sensitivity and specificity at the selected ROC threshold (Youden index) using the formulas:

LR + = Sensitivity / (1 − Specificity).

LR − = (1 − Sensitivity) / Specificity.

Posterior probabilities were then calculated using odds-form Bayes’ theorem:

Prior odds = p / (1 − p) Posterior odds = Prior odds × Likelihood Ratio.

Posterior probability = Posterior odds / (1 + Posterior odds).

Sensitivity analyses were performed in patients with available high-risk HPV data. Multivariable logistic regression models were adjusted for age and high-risk cytology, with variables selected based on clinical relevance and prior literature. To minimize overfitting, the number of predictors was restricted relative to the number of events. Correlations between key variables were assessed prior to model construction, and no relevant multicollinearity was identified. Additional sensitivity analyses incorporating high-risk HPV status and comorbidity variables (diabetes mellitus, hypertension, BMI, and smoking status) were conducted to evaluate potential confounding. Due to missing data in some covariates, extended models were interpreted with caution.

All statistical tests were two-sided, and *p*-values <0.05 were considered statistically significant.

## 3 Results

### 3.1 Patients’ characteristics

Table 1. provides a summary of the participants’ attributes in the study. By comparing data from three centers, a total of 1.142 conization procedures were analyzed. The median age of the patients was 38 years (interquartile range: 32–45 years). The median body mass index was 22.84 kg/m^2^ (interquartile range: 20.73–25.81 kg/m^2^). The study cohort had a median NLR of 1.79 (IQR: 0.62–7.88), a median PLR of 8.4 (IQR: 3.11–30.52), and a median LMR of 4.48 (IQR: 1.63–21.23). A total of 65% of all patients reported being non-smokers. In terms of HPV-status, high-risk HPV was detected in 64.8% of all patients. High-risk HPV status was available in 785 patients (68.7%). Among tested individuals, 94.3% were HPV-positive. When cytology was dichotomized into low- and high-risk categories, high-risk HPV positivity was not significantly associated with cytological risk group (*p* = 0.702). However, HPV positivity was significantly associated with histological grade (*p* < 0.001).

**Table 1:** Characteristics of the sample.

Characteristics (*n* = 1,142)	*N* (Range or %)
Total	1,142
Median age (years)	38 (32–45)
Median BMI (kg/m^2^)	22.84 (20.73–25.81)
Smoking status	
Yes	199 (17%)
No	742 (65%)
Not Reported	201 (18%)
Hr-HPV positivity	740 (64.8%)
Cytology results	1,100
Grade I	35 (3.1%)
Grade II	186 (16.3%)
Grade III	858 (75.1%)
Grade IV	21 (1.8%)
Conization results	1,124
Grade I	197 (17.3%)
Grade II	100 (8.8%)
Grade III	774 (67.8%)
Grade IV	53 (4.6%)
Median NLR	1.79 (0.62–7.88)
Median PLR	8.4 (3.11–30.52)
Median LMR	4.48 (1.63–21.23)

Categorical data are shown as frequencies (*n*), and continuous data are summarized as median values with interquartile ranges.

Patients were divided into four groups based on their cervical cancer screening results. 35 patients (3.1%) were categorized as Grade I (negative), 186 (16.3%) as Grade II (LSIL, ASC-US), 858 (75.1%) as Grade III (HSIL, ASC-H, AGC, AIS), and 21 (1.8%) as Grade IV (cancer). Similarly, the histopathological outcomes of conization were grouped into four distinct grades: 197 (17.3%) were Grade I (negative), 100 (8.8%) were Grade II (LSIL, ASC-US), 774 (67.8%) were Grade III (HSIL, ASC-H, AGC, AIS), and 53 (4.6%) were Grade IV (cancer).

### 3.2 Evaluation of cytology accuracy

In 1084 cases, data were available for both cytological examination and post-conization histological assessment. Comparison of these modalities revealed a clinically relevant discordance, with cytology underestimating the histological diagnosis in 13.93% of cases (151/1084; 95% confidence interval (CI) 12.0–16.1) and overestimating disease severity in 20.85% (226/1084; 95% CI 18.54–23.37).

**Figure 2:**
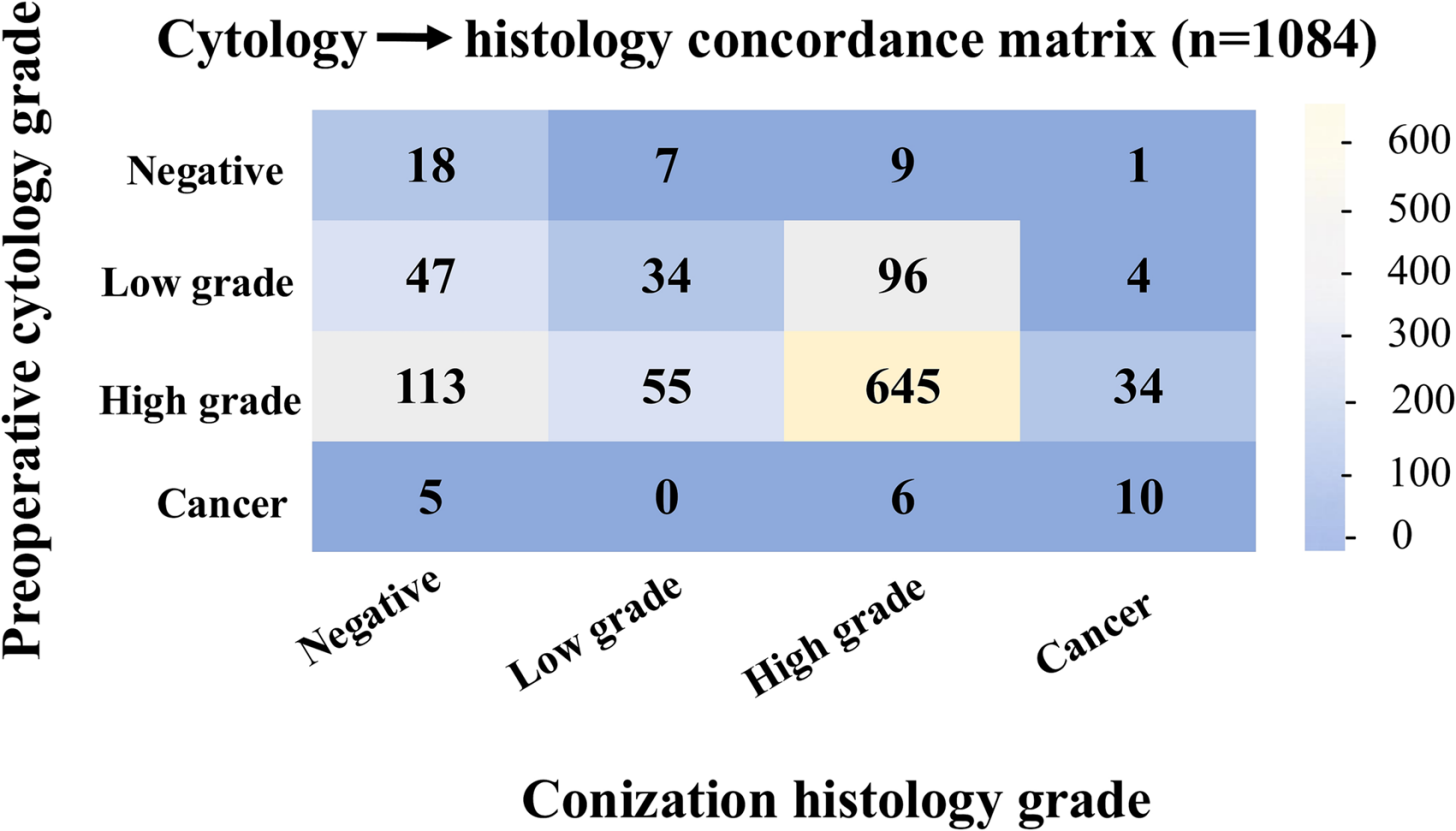
Concordance matrix comparing cytological and histological diagnoses. Clinically, this highlights the proportion of cases where cytology under- or overestimated histological severity, emphasizing the limitations of cytology alone in detecting invasive disease.

Focusing specifically on invasive disease, 49 cases were identified in which histology confirmed invasive cancer and a preoperative cytological result was available. Cytology correctly predicted the presence of invasive cancer in only 20.4% of these cases (10/49), while failing to do so in 79.6% (39/49). Accordingly, cytology demonstrated very low sensitivity for the detection of invasive cancer (sensitivity 20.4%), despite high specificity (98.9%) and a high negative predictive value (96.3%), with a positive predictive value of 47.6% (Figure 2).

### 3.3 Relationship between laboratory parameters and cytology results

**Figure 3:**
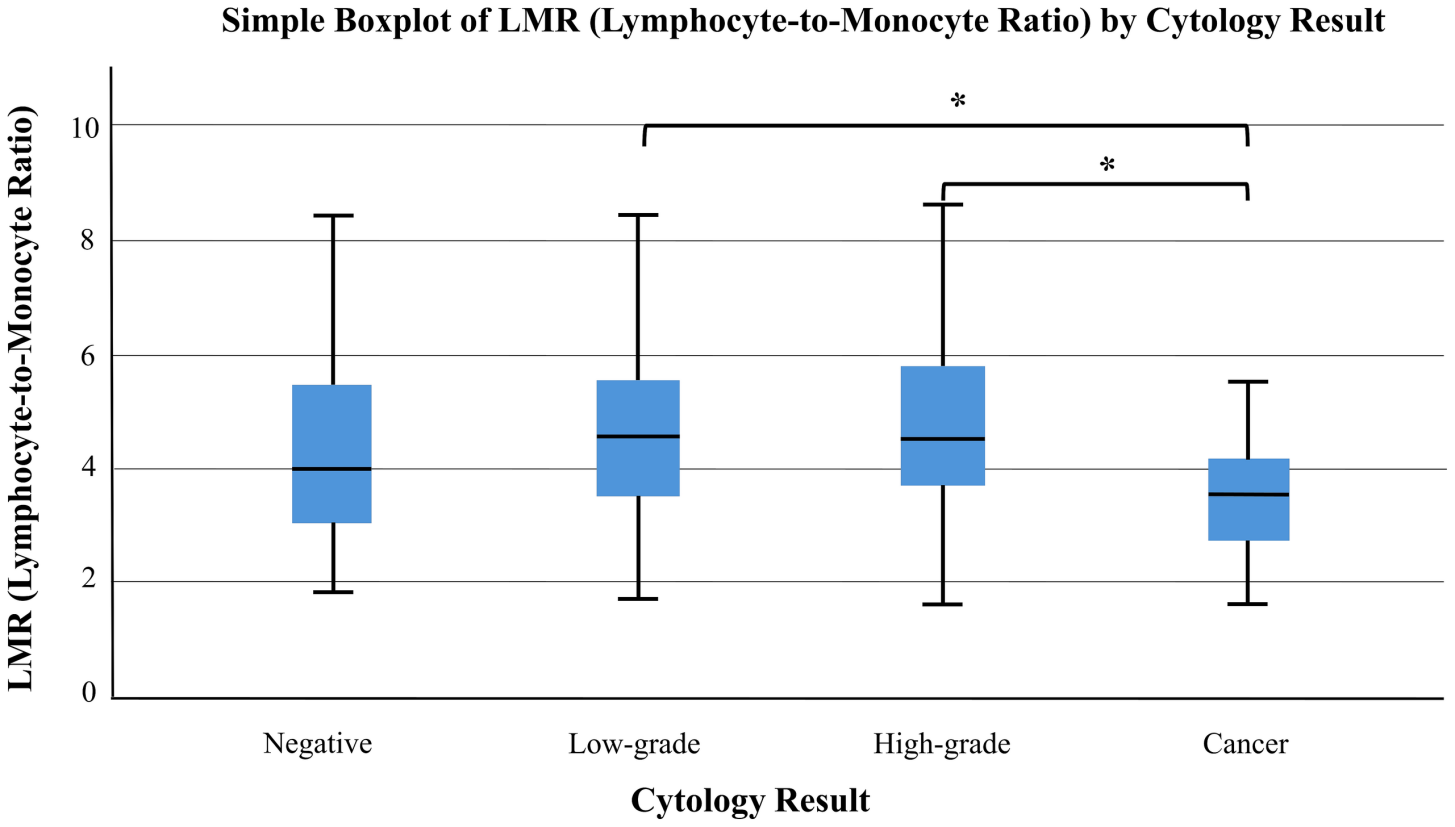
Distribution of lymphocyte-to-monocyte ratio (LMR) across different cytology result groups. A statistically significant difference in LMR was observed between the cytology categories (Kruskal–Wallis test, *p* = 0.0009). Box plots show median values and interquartile ranges; whiskers represent minimum and maximum values excluding outliers. Asterisks (*) denote statistically significant differences between groups. Lower LMR may be associated with higher-grade lesions.

The relationship between hematological parameters and cytology results was assessed using the Kruskal–Wallis test. No significant differences were observed in neutrophil-to-lymphocyte ratio (NLR) (*p* = 0.423) or platelet-to-lymphocyte ratio (PLR) (*p* = 0.7674) across the different cytological categories. However, a statistically significant difference was found in the lymphocyte-to-monocyte ratio (LMR) between cytology groups (*p* = 0.0009), suggesting that LMR may be associated with cytological findings (Figure 3.). Dunn’s *post hoc* test with Bonferroni correction revealed a significant difference in LMR between low-grade and cancer (*p* = 0.0223) and between high-grade and cancer (*p* = 0.0031) (Figure 3).

### 3.4 Relationship between laboratory parameters and histology results after conization

**Figure 4:**
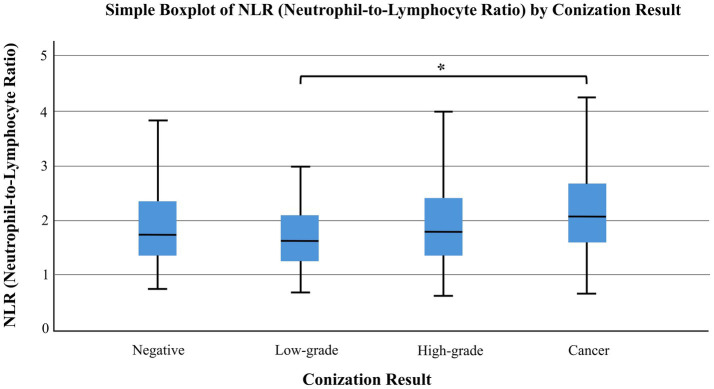
Neutrophil-to-lymphocyte ratio (NLR) values across different conization result groups. A statistically significant difference in NLR was observed between conization groups (Kruskal–Wallis test, *p* = 0.0178). Box plots display median values and interquartile ranges, with whiskers representing minimum and maximum values excluding outliers. An asterisk (*) denotes statistically significant differences between groups. Elevated NLR may indicate invasive histological disease.

**Figure 5:**
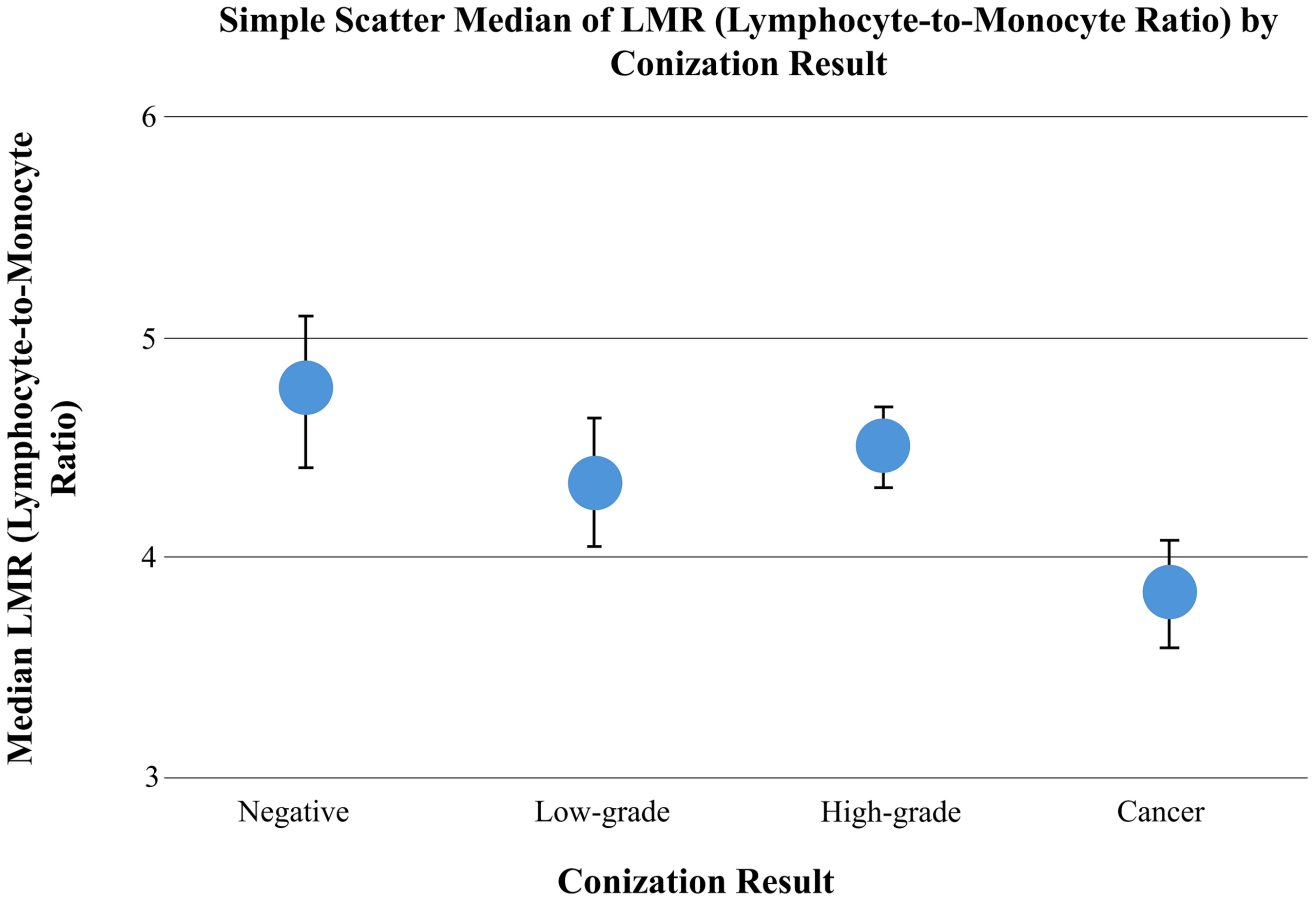
Median lymphocyte-to-monocyte ratio (LMR) across conization result groups. No statistically significant differences in LMR were observed between groups (Kruskal–Wallis test, *p* = 0.0763); however, a trend toward differences across increasing severity categories is apparent. The scatter plot displays group median LMR values with 95% confidence intervals, illustrating this trend. This trend may reflect changes in systemic immune balance across lesion severity.

The relationship between hematological parameters and histological outcomes of conization was evaluated using the Kruskal–Wallis test. A statistically significant difference in neutrophil-to-lymphocyte ratio (NLR) was observed across conization result groups (*p* = 0.0178) (Figure 4), while no significant differences were found for platelet-to-lymphocyte ratio (PLR) (*p* = 0.1803) or lymphocyte-to-monocyte ratio (LMR) (*p* = 0.0763). However, a trend-level difference was noted in LMR (Figure 5). Subsequent Dunn’s post hoc test with Bonferroni correction identified a significant difference in NLR between the low-grade lesion group and the cancer group (*p* = 0.0127), suggesting a potential role of NLR in distinguishing between benign and malignant histological findings.

When histological results were grouped and analyzed according to the presence of cancer, and comparisons were performed using the Mann–Whitney U test, statistically significant differences were observed between the cancerous and noncancerous groups in both NLR (*p* = 0.032) and LMR (*p* = 0.015), while a trend-level association was identified for PLR (*p* = 0.099) between these groups (Table 2).

**Table 2:** Relationship between laboratory parameters and histological results (cancerous vs. non-cancerous groups).

Hematological parameter	Mann–Whitney U	*z*	*p*
NLR	22526.5	−2.150	0.032*
LMR	21297.0	−2.437	0.015*
PLR	22259.5	−1.651	0.099

The table presents the results of the Mann–Whitney U test assessing the association between histologically confirmed cancer status and hematological parameters, including the neutrophil-to-lymphocyte ratio (NLR), lymphocyte-to-monocyte ratio (LMR), and platelet-to-lymphocyte ratio (PLR). *p < 0.05.

### 3.5 Diagnostic performance

**Figure 6:**
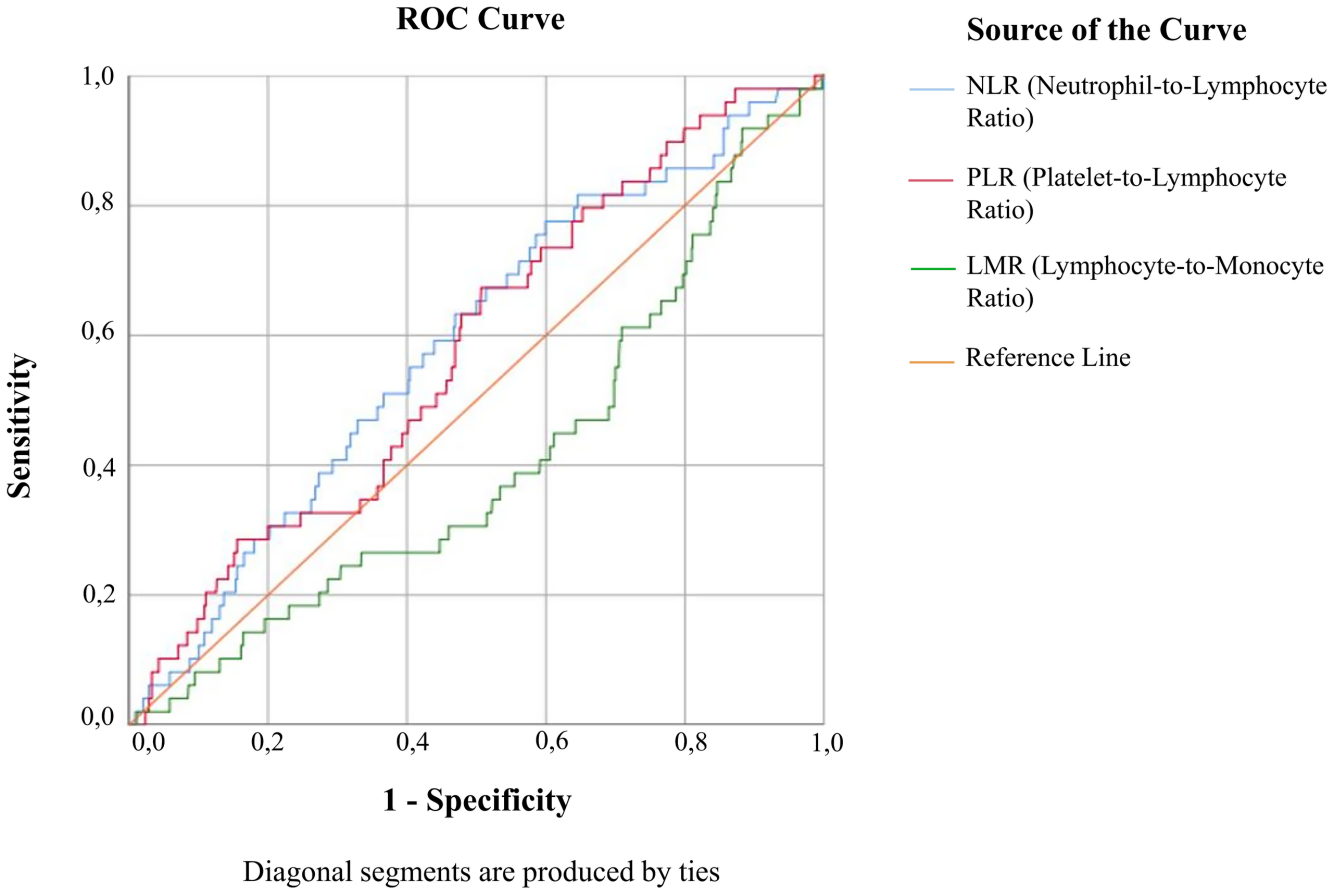
The receiver operating characteristic (ROC) curve compares the diagnostic performance of the neutrophil-to-lymphocyte ratio (NLR), platelet-to-lymphocyte ratio (PLR), and lymphocyte-to-monocyte ratio (LMR) for identifying cervical cancer. Sensitivity (true positive rate) is plotted on the *y*-axis, while 1-specificity (false positive rate) is on the *x*-axis. These markers demonstrate limited discriminative ability alone, but may be useful as rule-out adjuncts in triage strategies.

ROC analysis was performed to evaluate the diagnostic utility of NLR, PLR, and LMR in predicting cancer (Figure 6; Table 3). NLR showed an AUC of 0.588 (95% CI: 0.511–0.665; SE = 0.039; *p* = 0.032), indicating weak discriminative ability. Using the Youden index, the optimal cut-off (1.588) yielded 76.9% sensitivity and 60.4% specificity, while the Closest Top Left method identified 1.823 (63.5% sensitivity, 47.1% specificity). Despite high negative predictive values (>96%), positive predictive values were low (<6.4%), suggesting limited utility for confirming disease but potential value for ruling it out. PLR demonstrated similarly weak performance (AUC = 0.569, 95% CI: 0.491–0.647, SE = 0.040, *p* = 0.040), with Youden and Closest Top Left cut-offs yielding modest sensitivity and specificity. Again, high NPVs (>96%) contrasted with low PPVs (<7%), indicating limited confirmatory value. LMR performed slightly better (AUC = 0.600, 95% CI: 0.522–0.678, SE = 0.040, *p* = 0.015), with optimal cut-offs of 4.472 (Youden) and 4.166 (Closest Top Left) producing moderate sensitivity and specificity. As with the other ratios, NPVs were high (>96%) while PPVs remained low (<7.4%), supporting their role as potential rule-out markers rather than definitive diagnostic tools.

**Table 3:** Cut-off values via the Youden index and the Closest Top Left method.

Hematological parameter	Cut-off	Sensitivity	1-Specificity	Positive predictive value (PPV)	Negative predictive value (NPV)	Positive likelihood ratio (+LR)	Negative likelihood ratio (−LR)	Accuracy
NLR								
Youden index method	1.588	0.769	0.604	0.059	0.972	1.273	0.583	0.414
Closest Top Left method	1.823	0.635	0.471	0.062	0.967	1.348	0.690	0.534
PLR								
Youden index method	8.317	0.660	0.504	0.060	0.968	1.310	0.685	0.504
Closest Top Left method	8.578	0.620	0.474	0.060	0.966	1.308	0.722	0.530
LMR								
Youden index method	4.472	0.712	0.485	0.069	0.972	1.470	0.560	0.525
Closest Top Left method	4.166	0.635	0.411	0.073	0.969	1.550	0.620	0.591

The table shows the different optimal cut-off values with the Youden index and the Closest Top Left method to assess the sensitivity and specificity of the NLR, PLR, and LMR.

To further explore the clinical relevance of NLR beyond ROC-based discrimination, patients were stratified into three risk groups (low NLR: <1.588, intermediate NLR: 1.589–1.823, high NLR: > 1.823) using the Youden index and closest top-left cut-off values. Cancer prevalence increased progressively across NLR categories, with rates of 2.8% (95% CI: 1.61–4.84) in the low-risk group, 4.7% (95% CI: 2.31–9.44) in the intermediate-risk group, and 6.25% (95% CI: 4.48–8.65) in the high-risk group.

**Figure 7:**
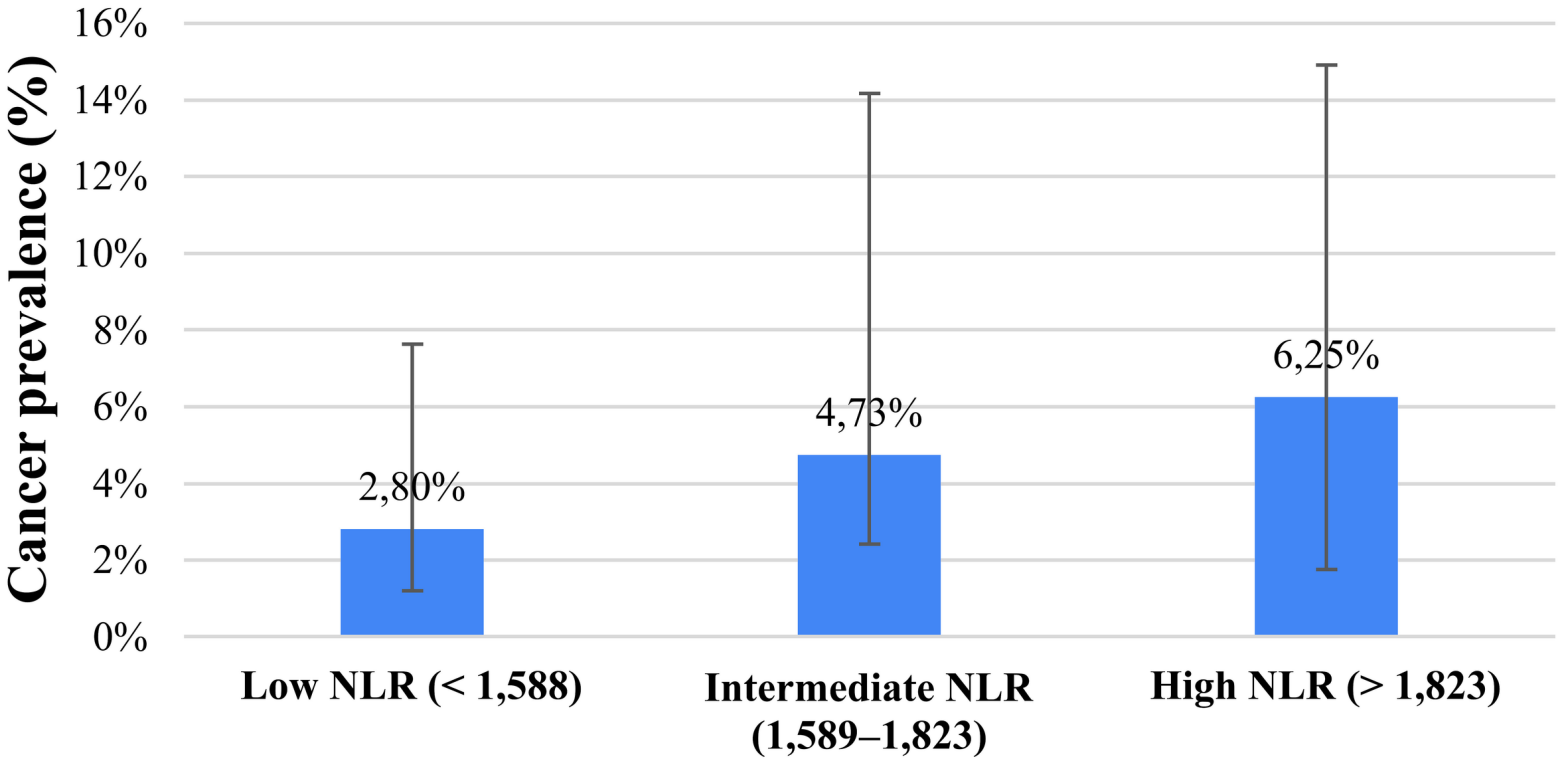
Cancer prevalence across NLR-based risk groups with 95% confidence intervals. Patients were stratified into low (<1,588), intermediate (1589–1823), and high (>1823) neutrophil-to-lymphocyte ratio (NLR) groups based on ROC-derived cut-off values. Cancer prevalence increased stepwise across NLR categories, from 28% in the low-risk group to 6.25% in the high-risk group. Error bars represent 95% Wilson confidence intervals. Higher NLR is associated with increased cancer prevalence.

This stepwise increase in prevalence supports a risk-stratification role of NLR, despite its modest overall discriminative performance (Figure 7).

**Figure 8:**
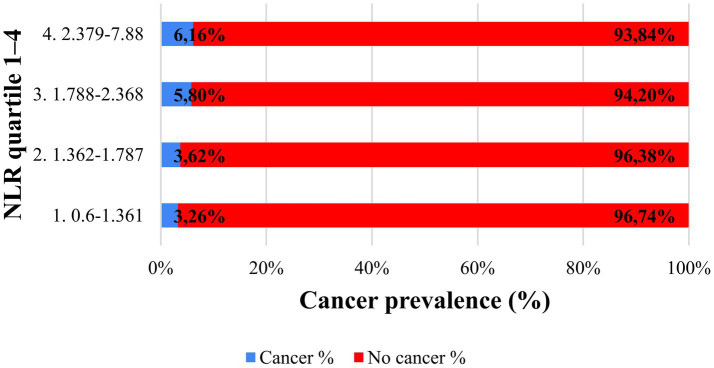
Cancer prevalence across NLR quartiles. Cancer prevalence (%) across quartiles of the neutrophil-to-lymphocyte ratio (NLR) in the study population. Patients were divided into four equally sized groups based on NLR values, and the proportion of patients with histologically confirmed cancer was calculated within each quartile. Cancer prevalence increased progressively from the lowest to the highest quartile, indicating a clear risk gradient. This supports the role of NLR as a graded risk marker.

To avoid reliance on arbitrarily optimized cut-off values, NLR was additionally analyzed in quartiles. Cancer prevalence increased progressively across quartiles, from 3.26% in the lowest quartile to 6.16% in the highest quartile, indicating a clear risk gradient. This supports the role of NLR as a risk stratification marker rather than a standalone diagnostic test (Figure 8).

### 3.6 Multivariable analysis and sensitivity analyses

Combining NLR, PLR, or LMR with cytology in multivariable logistic regression models modestly improved discrimination compared to cytology alone: AUC increased from 0.549–0.561 (cytology-only) to 0.623 (NLR), 0.635 (LMR), and 0.611 (PLR). In the model including NLR above cutoff and high-risk cytology, elevated NLR was independently associated with invasive cancer (OR = 2.04, *p* = 0.036), while high-risk cytology showed a borderline association (OR = 2.31, *p* = 0.080). Nagelkerke R^2^ was 0.025. Bayesian updating indicated that in high-risk cytology patients, a low NLR reduced post-test cancer probability from 5.15 to 2.97%, whereas a high NLR increased it to 6.56%.

Age was significantly associated with invasive cancer (OR 1.06 per year increase, *p* < 0.001), but its correlation with NLR was weak (Spearman *ρ* = 0.15). In models adjusted for age and cytology (*n* = 1,067), NLR remained directionally associated with invasive cancer (OR 1.17, 95% CI 0.89–1.55), although statistical significance was attenuated. Additional sensitivity analyses including diabetes or hypertension did not materially alter the magnitude or direction of NLR effect estimates.

BMI and smoking status were not significantly associated with invasive cancer (*p* = 0.974 and *p* = 0.101, respectively) and were excluded from final models. Overall, these results highlight the limited but consistent incremental value of inflammatory markers, particularly NLR, as complementary risk stratification tools rather than standalone diagnostic tests.

## 4 Discussion

This multicenter study provides a comprehensive evaluation of cytology–histology concordance and the potential added value of systemic inflammatory markers in the triage of cervical lesions. Although global health initiatives, such as the World Health Organization’s cervical cancer elimination strategy aim to reduce the burden of disease, cervical cancer remains a significant challenge, particularly in resource-limited settings (6, 13).

In our cohort, cytology–histology discordance was clinically meaningful, with cytology underestimating disease severity in nearly 14% of cases and, most critically, failing to identify approximately 80% of histologically confirmed invasive cancers. Consequently, cytology demonstrated very low sensitivity (20.4%) for invasive cancer, despite high specificity (98.9%) and a high negative predictive value (96.3%).

These findings underscore a central paradox of cytology-based triage: while negative results are reassuring, positive identification of invasive disease is unreliable (14). Accordingly, cytology functions primarily as a rule-out tool rather than a rule-in test for cancer, reinforcing the need for complementary triage strategies.

Recent evidence has increasingly highlighted the intrinsic limitations of cytological sampling and interpretation, supporting the incorporation of additional biomarkers into cervical cancer screening and triage algorithms (15, 16).

In line with WHO recommendations, high-risk HPV testing is expected to become the primary screening modality in many countries (6). However, HPV-based screening still requires effective secondary triage to manage high-risk results and avoid unnecessary diagnostic escalation. This is particularly important in settings where access to colposcopy or advanced molecular testing is limited (17). From a clinical perspective, complete blood count (CBC)-derived inflammatory markers are not intended to replace established triage strategies such as high-risk HPV testing, dual-stain cytology (p16/Ki-67 immunocytochemistry), or colposcopic evaluation. Rather, their potential value lies in providing inexpensive, widely available adjunctive information that may complement existing risk stratification approaches. In particular, in settings where access to molecular biomarkers, dual-stain testing, or advanced imaging is limited, systemic inflammatory markers could contribute to preliminary risk assessment or help support conservative management decisions in patients with otherwise equivocal findings. In contemporary cervical cancer prevention pathways, HPV testing serves as the primary screening modality, while cytology or molecular markers guide secondary triage. Within this framework, CBC-based indices may function as supportive markers rather than primary decision tools.

Given the central role of high-risk HPV in cervical carcinogenesis, we performed sensitivity analyses to assess its potential confounding effect on the association between inflammatory markers and invasive disease. Adjustment for high-risk HPV status did not materially alter effect estimates, suggesting limited confounding in this cohort. The reduction in statistical significance observed in some models was primarily attributable to reduced sample size due to missing HPV data. Consistent with this, restriction to HPV-positive individuals—given the high prevalence of HPV—did not improve model discrimination.

The investigation of CBC-based biomarkers in oncology is grounded in the observation that persistent, low-grade inflammation is a hallmark of the tumor microenvironment (18, 19).

NLR, PLR, and LMR have demonstrated prognostic relevance across multiple malignancies, including osteosarcoma (7), non-small cell lung cancer (19), and endometrial cancer (20).

Among these markers, the neutrophil-to-lymphocyte ratio (NLR) is the most extensively studied inflammatory index in cervical cancer. Elevated pretreatment NLR values have been associated with HPV presence, advanced stage, lymph-node involvement, poorer treatment response, and reduced survival (21, 22). In a large cohort of 460 surgically treated cervical cancer patients Zhang et al. reported that higher preoperative NLR correlated with adverse histopathologic features and independently predicted poorer progression-free survival, whereas PLR did not retain independent prognostic value (23). In our study, significant association was observed between NLR and histopathological findings following conization, particularly differentiating low-grade lesions from invasive cancer. This suggests that NLR may reflect biological features of tumor invasiveness rather than cytomorphological severity alone. Our findings are consistent with those of Vida et al., who observed higher NLR values in patients with more severe histopathological outcomes (24). Importantly, in multivariable analysis that included high-risk cytology, elevated NLR remained independently associated with invasive cancer, whereas cytology alone showed only borderline significance. Although the model’s overall explained variance was modest, this result indicates that NLR provides biological risk information that is not fully captured by cytological assessment alone. The relatively low Nagelkerke R^2^ values indicate that the included variables explain only a limited proportion of the variability in invasive cancer risk. This is consistent with the multifactorial nature of cervical carcinogenesis and supports the interpretation of these markers as complementary rather than standalone predictors. Age, in turn, emerged as a strong independent predictor of invasive cancer. Adjustment for age and available comorbidities somewhat attenuated the statistical significance of NLR, yet effect estimates remained directionally consistent, suggesting that major confounding by these factors is unlikely. Nonetheless, the relatively low number of invasive cancer events limits statistical power, and these findings should be interpreted with caution.

We observed statistically significant differences in LMR between low-grade and cancer groups, as well as between high-grade and cancer groups, suggesting that LMR may reflect changes in systemic immune balance across disease severity (25). Szabó et al. similarly reported significant variation in LMR across cytological categories, supporting its association with cytological progression (26). Together, these findings suggest that LMR may be more closely linked to cytological phenotype and baseline immune status, whereas NLR appears to align more strongly with definitive histological invasiveness (27). This distinction may explain the divergent behavior of these markers across cytology- and histology-based analyses. While Kalas et al. reported an association between elevated PLR levels and malignant histopathological outcomes (10), our study found no significant link between pretreatment PLR values and either cytological or histopathological results. The discrepancy between findings may reflect both methodological and cohort-related differences. In the present study, PLR was calculated using lymphocyte percentage rather than absolute lymphocyte count. This differs from the conventional definition commonly used in the oncology literature and may limit direct comparability with previously published thresholds. Moreover, our substantially larger cohort reduces the likelihood of chance findings and non-reproducible associations, potentially contributing to the absence of a significant effect in our analysis. Further prospective studies using standardized PLR definitions and harmonized methodologies are warranted to clarify these inconsistent observations.

Although ROC analyses demonstrated poor overall discriminatory performance for NLR, PLR, and LMR (AUC 0.57–0.60), this does not negate their potential adjunctive value. In low-prevalence settings such as cervical cancer triage, negative predictive value (NPV) is strongly influenced by disease prevalence, and high NPVs may provide practically useful reassurance. Across multiple cut-off strategies, all three markers showed consistently high NPVs (approximately 0.97), despite low PPVs and weak AUC values. Similar observations have been made for established cervical screening tools, where clinical utility is primarily derived from effective rule-out performance rather than confirmatory accuracy (28). In this context, CBC-derived inflammatory ratios may help identify patients with a very low probability of invasive disease. This may improve triage efficiency despite limited standalone diagnostic performance. However, because the present cohort consisted exclusively of women undergoing conization, the study population was inherently enriched for higher-risk lesions, which may influence the estimated diagnostic performance metrics and limit the direct generalizability of these findings to broader screening populations.

Beyond ROC-based evaluation, stratified and Bayesian analyses provided additional clinically relevant insights. Cancer prevalence increased progressively across NLR-based risk strata, indicating a reproducible risk gradient. Bayesian probability updating further demonstrated that NLR predominantly influences post-test probability in the negative direction. In patients with high-risk cytology, a low NLR reduced post-test cancer probability by approximately 40%, whereas a high NLR produced only a modest increase. Nevertheless, the absolute change in post-test probability remained relatively modest, and these results should therefore be interpreted cautiously. Bayesian updating with NLR does not provide decisive diagnostic shifts. Instead, it offers incremental refinement of risk estimates that may support clinical judgment within a multimodal triage framework.

Importantly, when inflammatory ratios were integrated with cytology in multivariable models, discrimination improved compared to cytology alone. Although the absolute AUC values remained in the weak-to-moderate range, the consistent incremental improvement observed across all three inflammatory ratios suggests that they provide complementary information beyond cytology. These findings further support their complementary role in triage.

Taken together, our findings identify cytology–histology discordance—particularly for invasive cancer—as a clinically relevant limitation in cervical cancer triage. CBC-derived inflammatory markers, especially NLR and LMR, provide biologically informative, low-cost adjunctive data but do not replace established diagnostic modalities. Despite limited discriminatory performance, their consistently high negative predictive value and independent association with invasive disease suggest potential utility in risk-adapted decision-making. Within a multimodal framework, these markers may support conservative management and more efficient triage, particularly in settings with constrained diagnostic resources. Prospective validation in larger, unselected populations is warranted.

### 4.1 Strengths and limitations

The major strengths of this study include its large, multicenter cohort, rigorous statistical methodology, and the use of multiple analytical approaches, including the Kruskal–Wallis test, Mann–Whitney U test, logistic regression, and ROC curve analysis to quantitatively evaluate the predictive performance of NLR, PLR, and LMR.

However, several limitations should be noted. As a retrospective study, it is subject to inherent selection and information biases. The cohort consisted exclusively of women already selected for conization, limiting generalizability to broader primary screening populations. Verification bias may have influenced the observed sensitivity of cytology, as histopathological confirmation was primarily performed in patients with prior abnormal cytology or clinical suspicion. Consequently, the cohort is enriched for higher-risk cases, and microinvasive cancers are likely underestimated. The discriminatory performance of the inflammatory markers was modest (AUC 0.57–0.60), only slightly above chance, suggesting minimal clinical separation between groups, and observed statistical significance may partly reflect the large sample size rather than clinically meaningful effect sizes. Importantly, the PLR calculation applied in this study was based on lymphocyte percentage rather than absolute lymphocyte count, which differs from the conventional definition most commonly used in the oncology literature. This methodological distinction may limit direct comparability with previously published PLR thresholds and should be considered when interpreting the results. Additional potential confounders, including comorbidities, concurrent medications, and demographic factors, were not fully controlled. Preoperative CBC sampling was conducted within one month prior to surgery in accordance with a standardized institutional protocol. No systematic differences in sampling timing were observed between outcome groups. Nevertheless, the allowable preoperative interval may have introduced limited biological variability in inflammatory indices, potentially attenuating effect estimates through non-differential measurement variability rather than systematic bias. Residual confounding by unmeasured systemic inflammatory or autoimmune conditions cannot be excluded in this retrospective cohort. In addition, as analyses were based on complete-case data, the potential for bias due to non-random missingness cannot be excluded, which may affect the robustness and generalizability of the multivariable models.

The relatively low number of invasive cancer cases (4.6% of the cohort; 53/1142 overall and 49 events included in the multivariable model) may limit the precision of regression-based effect estimates and contribute to wider confidence intervals, necessitating cautious interpretation of multivariable results. Although the model was intentionally restricted to a limited number of predictors to reduce the risk of overfitting, the modest absolute event count may still affect the stability and robustness of the estimates.

Furthermore, although these markers are unlikely to serve as effective standalone screening or triage tools in the general population, they may still provide meaningful information when incorporated into multivariable prediction models alongside other clinical, demographic, or laboratory factors. In such composite models, NLR, PLR, and LMR could contribute complementary biological insights, enhance risk stratification, and help identify clinically relevant subgroups, providing a rationale for further prospective investigation.

### 4.2 Implication for practice

Owing to their low cost, accessibility, and ease of calculation from routine blood tests, NLR and LMR have potential as supplementary biomarkers in the assessment of cervical lesions. While they are not intended to replace cytological or histological evaluation, these markers may provide supportive guidance for patient triage, particularly in settings with limited access to advanced diagnostic tools. In the future, NLR and LMR could assist clinical decision-making by identifying individuals who may benefit from closer monitoring or additional testing, thereby improving risk stratification and optimizing the use of healthcare resources across both high- and low-resource environments.

## Data Availability

The raw data supporting the conclusions of this article will be made available by the authors, without undue reservation.
